# Parliament and Publications

**Published:** 1911-03-25

**Authors:** 


### Parliament and Publications.

### NURSES' REGISTRATION.

Mr. Munro Ferguson has introduced, in the House of Commons, a Bill to regulate the qualifications of trained nurses and to provide for their registration. The Bill has not yet been printed.

### REPORT OF THE PLAGUE IN LONDON.

In reply to Mr. Lonsdale, Mr. John Burns stated in the House of Commons that there was no foundation for the statement that cases of plague had recently occurred in London.

### RAT VIRUS.

Mr. Burns states that he is not aware of any recent case of illness attributed to virus sold for the destruction of rats and other vermin.

### ILLEGAL OPERATIONS.

In addressing the grand jury at Manchester Assizes, Mr. Justice Grantham said the offence of illegal operation seemed to be more rife in Lancashire than in other parts of the country. As illustrating the extent to which the offence is carried on, his Lordship said he remembered a case in Manchester where a man, convicted of the offence, who had been in the habit of charging a fee of half-a-sovereign for his services, was found to have 147 half-sovereigns in a drawer, showing that he had conducted at least that number of operations. In passing sentence of 18 months' imprisonment on a woman who pleaded "guilty" to having used an illegal instrument, the judge said if it were not for women like the prisoner and other horrible creatures who got good incomes by this trade, the crime would not be committed.

### VACCINATION AND SMALLPOX.

A number of questions have been asked in the House of Commons with reference to vaccination and the outbreak of smallpox in the East End. It appears from the replies of Mr. John Burns that of 44 patients six are five years of age or under, and of these three have not been vaccinated, three were vaccinated in infancy; two have been re-vaccinated since the onset of the disease. Seven are between five and fifteen years, of whom one has not been vaccinated, five were vaccinated in infancy, one case is doubtful; four cases have been vaccinated since the onset of the disease. Fifteen are between fifteen and thirty, of whom two have not been vaccinated, twelve were vaccinated in infancy, one is doubtful; eight of these have been re-vaccinated since the onset of the disease. Sixteen are cases of persons over thirty, of which fourteen were vaccinated in infancy; two cases are doubtful; ten cases have been vaccinated since the onset of the disease. During the first half of last year 456,533 births were registered, and 110,851 exemption certificates were received.

### THE ARMY ESTIMATES AND THE MEDICAL SERVICE.

The Army Estimates for 1911-12 have been issued. The sum which will be required in the year ending March 31, 1912, to defray the expense of the pay, &c., of the medical establishment and of medicines, under Vote 2 is estimated at £437,000. This is a decrease on the previous year of £15,000, due to increased provision for medical equipment, to the transfer to Vote 4 (Territorial services) of certain medical officers to be employed with the Territorial Force, and to the operation of the system of time promotion, under which the average rank and rate of pay of officers will be lower than in 1910-11. The total provision for medical and hospital services is £1,457,000, allotted as follows: (1) Medical establishment (officers £582,490, W.O., N.C.O., and men £331,440, nurses, civilians, &c., £98,605), £1,012,535; (2) R.A.M.C., Special Reserve, £19,010; (3) medical establishment of the Territorial Force, £139,555; (4) Medical Staff at War Office, Educational Establishments, &c., £26,700; (5) medicines, instruments, &c., £20,400; (6) Barrack services for hospitals, £24,500; (7) Hospital provisions, medical comforts, &c., £99,000; (8) Fuel, light, and water for hospitals, £22,500; (9) Hospital clothing, £4,600; (10) Hospital stores and field hospital equipment, £7,250; (11) Works services, £80,950. The value of ordinary rations not issued while men are in hospital is about £34,500. About £60,000 is deducted from pay as "hospital stoppages," and messing allowance to the value of £26,000 is not issued.—"Army Estimate" (53), price 1s. 10d.

### DEATHS FROM CHICKEN-POX.

The numbers of deaths certified as resulting from chicken-pox of children under fifteen years of age in England and Wales were 93 in 1908 and 94 in 1909. Experience has not shown, said Mr. Burns, answering a question asked by Mr. Crawshaw Williams, that there is any evidence that any considerable proportion of the recorded mortality from chicken-pox is really due to smallpox.

### CERTIFYING SURGEONS AND FACTORIES.

The report of the Departmental Committee on Accidents in Places under the Factory and Workshop Acts contains some recommendations with regard to the duties of certifying surgeons. There appears to be considerable variation in the efficiency with which the certifying surgeons' work of reporting on accidents is carried out, both as regards the thoroughness of their investigation and report, but it is clear that many of them bring to it much interest and enthusiasm. The Committee consider that, quite apart from any consideration of the efficiency with which the work is done, the time has come when much of the work could be dispensed with. The Committee attach great importance to the examination of children and young persons for the purpose of eliminating the unfit from factory work. In order to facilitate and improve the work of certifying surgeons, steps might be taken to bring them into closer touch with the inspectors, and the Committee suggest that conferences at certain intervals between inspectors and surgeons would be useful.—"Accidents Committee," Cd. 5535, price 7d.

### INFANT MORTALITY AND THE NOTIFICATION OF BIRTHS.

The Notification of Births Act has been put in force in 271 areas of local government, and it is significant that the average infant death-rate in nearly all those districts in 1909 was appreciably lower than in 1905. In Grimsby, for instance, the infant death-rate fell from 174 per 1,000 births in 1905 to 118 in 1909; in Bolton from 167 to 128; in Dudley from 167 to 140; in Halifax from 131 to 97; in Ipswich from 144 to 92; in Kingston-on-Hull from 152 to 114; in Lincoln from 148 to 82; in Merthyr Tydfil from 193 to 143; in Norwich from 174 to 119; in Oldham from 150 to 119; in Rochdale from 133 to 104; in Sheffield from 167 to 118; in Southampton from 132 to 106; in Stockport from 168 to 132; in Worcester from 154 to 101; in York from 129 to 99; in Barrow-in-Furness from 135 to 81; in Canterbury from 119 to 73; in Hastings from 113 to 79; in Oxford from 116 to 75; and in West Hartlepool from 146 to 113. On the other hand, the death-rate of infants under one year of age is higher in four places where the Act is in force; thus in St. Helens the rate has risen from 134 to 150; in Swansea from 131 to 159; in Wolverhampton from 137 to 138; and in Bournemouth from 83 to 100. In considering these figures it must, of course, be borne in mind that the infant death-rate for the whole country has also fallen considerably.

